# Innate Immunity Mediated Inflammation and Beta Cell Function: Neighbors or Enemies?

**DOI:** 10.3389/fendo.2020.606332

**Published:** 2021-02-08

**Authors:** Antonio Citro, Francesco Campo, Erica Dugnani, Lorenzo Piemonti

**Affiliations:** ^1^ San Raffaele Diabetes Research Institute, IRCCS Ospedale San Raffaele, Milan, Italy; ^2^ Università Vita-Salute San Raffaele, Milan, Italy

**Keywords:** type 1 diabetes, inflammation, cytokines, innate immunity, insulin secretion

## Abstract

Type 1 diabetes (T1D) is still considered a huge burden because the available treatments are not effective in preventing the onset or progression of the disease. Recently, the idea that diabetes is an autoimmune disease mediated exclusively by T cells has been reshaped. In fact, T cells are not the only players with an active role in beta cell destruction. Macrophages and neutrophils, which physiologically reside in pancreatic tissue, can also participate in tissue homeostasis and damage by promoting innate immune responses and modulating inflammation. During the development of the pancreatic islet inflammation there is a strong interplay of both adaptive and innate immune cells, and the presence of innate immune cells has been demonstrated both in exocrine and endocrine pancreatic compartments during the earliest stages of insulitis. Innate immune cell populations secrete cytokines, which must be considered both as physiological and pathological mediators. In fact, it has been demonstrated that cytokines could regulate directly and indirectly insulin secretion and, simultaneously, trigger inflammatory reaction. Indeed, cytokines pathways could represent targets both to improve glucose metabolism and to prevent autoimmune damage. Concordantly, the combination of immunomodulatory strategies against both innate and adaptive immunity should be tested in the next future, as they can be more efficient to prevent or delay islet damage and T1D onset.

## Introduction

Although the available options of antidiabetic drugs are considerably increased and the burden of diabetes management is decreased, still we lack of therapies able to stop the pathological process that results in β cell failure and destruction. As of now, in the therapeutic clinical arsenal there are no registered treatments with indication for preventing or curing the onset or progression of type 1 diabetes (T1D). T1D was previously depicted as an exclusively T cell-mediated autoimmune disease with an inflammatory component, in which cells of specific immunity play a crucial role in β cell destruction. More recently, the immunological pathogenesis of T1D and in general of autoimmune diseases has been significantly reshaped, proposing a more complex interaction between several immunological players of the adaptive and innate immunity. In this context, the pancreatic islet inflammation present in T1D could result from an interaction between resident innate immune cells and target β cells (1). Moreover, it has been recognized that β cells proteins are released, taken up, processed, and presented by innate immune cells to islet auto-reactive T cells after their migration to the pancreatic draining lymph nodes (2). This cascade mediates the recruitment of activated T cells that increases the initial damage by mediating β cell killing and promotes further inflammation, generating a vicious loop. In this review, we will recapitulate the recent advances in understanding of the inflammation mediators and their implication in the beta cell function, paying particular attention to the relationship between innate immunity and insulin secretion both in pathological and physiological conditions.

## Innate Immune and Beta Cells: The Favorite Pancreatic Roommates

### Macrophages

It is widely described that innate immune cells (i.e., neutrophils, monocytes, and macrophages) populate the pancreatic endocrine compartment (3). Indeed, macrophages reside in the healthy mouse islets beginning in the perinatal stages. They represent more than 80% of the intra islet immune cells (3–6) and some studies suggested that these macrophages are involved in islet morphogenesis (4). Endocrine resident macrophages derive from definitive hematopoiesis and are strongly self-maintained by local proliferation. Pancreatic macrophages showed different phenotype in relation to developmental stages, anatomical location, or metabolic profile. The immunoprofiling of pancreatic resident macrophage show the presence of F4/80^lo^CD11c^+^ within the islet structure whereas F4/80^hi^CD11c^–^ macrophages largely reside in the peripheral islet area. The distinct role of these two different macrophage subpopulations is still incomplete and more studies are needed to understand how pancreatic macrophages participate in tissue homeostasis maintenance and function of β cells (7). They did not reflect the classical M1 or M2 classification that is well described in other tissues.

Since their discovery, the role of these cells remains unclear. They are an essential player during early life development to establish a functional beta cell mass (6). Recent evidences structurally mapped the resident macrophages in the pancreas, showing their role as sentinel cells in the peri-islet structure. Two-photon microscopy of murine islets showed that resident macrophages are in close proximity of pancreatic blood vessels with extensive filopodial activity. Moreover, within the islet, macrophages are in intimate contact with beta cells. Through this cell contact (the beta cell–macrophage “synapse”) the macrophages may take up insulin-containing vesicles. Functional studies in NOD mice showed that resident macrophages, thanks to their long filopodia, can dynamically probe whole islet volume including the vessel lumen and present insulin peptides to insulin-reactive T cells (8). Moreover, these cells may be activated from blood products and are able to monitor the islet secretory activity by detecting ATP endogenous levels through their purinergic receptors (9, 10).

### Neutrophils

By combining single-cell RNA-Seq and immunophenotypic analyses to build a comprehensive view of the pancreatic mouse innate immunity cell landscape, a tissue-specific cell heterogeneity in myeloid cell (including monocytes, neutrophils, dendritic cells, and macrophages populations) was described and putative gene networks underlying the pancreatic specialization were identified (11). This is in line with past literature and confirms the presence of resident neutrophils in the healthy pancreatic tissue. As an important element of the inflammatory response, neutrophils can direct and guide the innate immune response by engaging in complex interactions with macrophages, natural killer cells, dendritic cells, and through crosstalk with most of the cellular effector mediators (12). After their activation, neutrophils can promote an innate immune response through releasing soluble pattern recognition molecules, which have the capacity to augment phagocytosis, stimulate complement, and modulate inflammation. They can also secrete a diversity of cytokines, neutrophil extracellular traps (NETs), and microorganism‐ and tissue‐damaging molecules to participate in innate inflammatory milieu. Pancreas-infiltrating neutrophils were recently observed in T1D patients by electron microscopy and immune-histochemical analysis mainly localized at the level of very small blood vessels in the exocrine pancreas (13). Neutrophils infiltrate the pancreas prior to the onset of the T1D symptoms and they continue to do so as the disease progresses (14). Of interest, a fraction of these pancreas-infiltrating neutrophils also extrudes NETs, suggesting a tissue-specific pathogenic role (14). These findings are in line with previous evidence in the NOD mouse model. Based on flow cytometry and immunohistochemistry on pancreatic infiltrating cells, we previously described the presence of neutrophils at different stages of the autoimmune disease (15). Moreover, we showed that the inhibition of the neutrophil recruitment, mediated by the CXCL8-CXCR1/2 pathway, might prevent and revert the hyperglycemia, suggesting the relevant role of those cells in the T1D onset and progression (15). Similarly, Diana et al. confirmed that the use of neutrophil neutralizing antibody in the asymptomatic stage of the disease could improve the T1D progression in NOD mouse (16). They suggested that β cell debris form immune complexes with dsDNA-specific IgGs secreted by B-1a cells. Neutrophils produce DNA-binding peptide that potentiates these immune complexes, inducing IFN-α secretion by pancreatic pDCs through TLR9.

## Can Immunological Stress Signals Affect Insulin Secretion?

### Insulin Secretion Mechanism

Although β-cells adapt well to changes in metabolic demand (whether acute or chronic, from starvation to over nutrition), persistently high insulin demand leads to progressive β cells dysfunction and loss (17). Plasma glucose concentration is the primary mediator of insulin secretion, but also a variety of other mediators (peptide hormones, ions, neurotransmitters, and pharmaceuticals) contribute in the maintenance of glucose homeostasis. Mounting evidences suggest that the components of the immune system (localized within islets or not) may also play a physiological role in β cell function. As early as 1992, the existence of immune-neuroendocrine interactions in controlling glycaemia was proposed, suggesting as mediators some specific cytokines like Interleukin-1 (IL-1), Tumor Necrosis Factor (TNF) alpha, and IL-6 (18). It was observed that administration of low doses of lipopolysaccharide, an inducer of several pro-inflammatory cytokines, caused a profound and long lasting hypoglycemia. Now it is well established that inflammatory drivers can prompt a modulation of insulin secretion sharing the same signaling mechanisms of glucose. An elevation of intracellular cytosolic Ca2+ ([Ca2+]i) is essential for glucose stimulated-insulin secretion (GSIS). In β cells, glucose increases glycolytic flux that enhances the ATP/ADP ratio and brings to closure of plasma membrane ATP-sensitive K+ channels; this leads to rapid depolarization and the opening of voltage-dependent L-type Ca2+ channels (VDCC) with consequent Ca2+ influx into the cell. The [Ca2+]i increase sustains exocytosis of readily releasable insulin secretory granules *via* SNARE and calcium-regulated proteins (priming, docking, and fusion phases) (19). A second pathway, resulting in the secretion of insulin granules, involves the activation of adenylyl cyclase and the subsequent increase of intracellular cAMP, activation of protein kinase A (PKA), and lastly the release of Ca2+ stored in endoplasmic reticulum. This cAMP/PKA-dependent pathway is the canonical signaling pathway of GLP-1 stimulated insulin secretion in the presence of glucose (20). In addition, the activation of protein kinase C (PKC)- phospholipase C (PLC) signal pathways evokes calcium mobilization and retains a role in insulin release in the presence of metabolic stimulus (20, 21).

### Immunological Stress Signals

Epidemiological evidences support the role of the viral infection as a possible environmental trigger for the development of T1D (22). As of now, it is not clear the exact mechanism behind the viral infection leading to T1D onset, but numerous studies evidence multiple possible mechanisms, including the initiation of the innate immune response. For example, the enteroviruses have shown specific islet cell tropism through detection of viral RNA and viral proteins in pancreatic sections of post-mortem T1D patients (23, 24). We investigated as well the potential role of the viral infection and innate immunity pathways in animal and human pancreatic tissues, paying particular attention to influenza virus (25): H1N1 and H3N2 and avian H7N1 and H7N3 influenza virus were able to infect a selection of human pancreatic cell lines and human pancreatic islets. In this context, the cytokine activation profile indicates a significant increase of innate immune mediators such as MIG/CXCL9, IP-10/CXCL10, RANTES/CCL5, MIP1b/CCL4, Groa/CXCL1, interleukin 8 (IL-8)/CXCL8, tumor necrosis factor alpha (TNF-α), and IL-6 (25). Overall these data suggested that influenza virus may play a role as a causative agent of pancreatitis and diabetes in humans and other mammals.

Also coxsackievirus B5 (CBV-5)-DS lytic strain has a tropism for pancreatic human islets, and it has been demonstrated that the expression of pro-inflammatory cytokine genes (IL-1α, IL-1 β. TNF-α, and TRAIL) mediated cytokine-induced beta cell dysfunction is correlated with the lytic potential of the virus (26). However, as we previously discussed, the innate immune molecules are also physiologically part of the pancreatic microenvironment, and here we describe their putative relevant roles in modulating the insulin secretion and in controlling the beta cell response.

Among cytokines, IL-1β was deeply studied as an insulin secretion modulator. A short-term exposure of β cells to IL-1β is able to potentiate the glucose-dependent insulin secretion in rodent and human islets by increasing granule trafficking and SNARE complex formation without affecting Ca2+ entry and insulin content (27, 28). Islets are enriched in expression of interleukin-1 receptor type I (IL-1R) and IL-1β plays a physiological role in promoting glucose homeostasis, as demonstrated using a model of pancreatic IL-1 receptor deletion (29). IL-1R^Pdx1−/−^ mice display a reduction of 25% in GSIS in isolated islets; similarly, *in vivo*, after an intraperitoneal glucose bolus, insulin secretion in IL-1R^Pdx1−/−^ mice was decreased by 56% in comparison with littermate controls (29). However, a chronic exposure to IL-1β impairs insulin secretion (30) and might result in β cell exhaustion. Recently it has been demonstrated that higher levels of circulating IL-1β correlate with higher fasting plasma glucose concentrations in healthy and in hyperglycemic diabetic individuals (31, 32). Accordingly, 48 hours of exposure of EndoC-βH1, a human beta cell lines, to pro-inflammatory cytokines IL-1β and IFN-γ reduced insulin secretion in GSIS in the presence of 20 mM glucose (33). Interestingly, these pro-inflammatory cytokines remodeled the β cell regulatory landscape (induction of a subset of novel and primed regulatory regions with a predominantly induction of gene transcription rather than transcript down-regulation) both in EndoC-βH1 and in human islets (33).

Regarding the cross talk between islets and inflammatory drivers, other cytokines deserve mention too. Interleukin 6 is a pleiotropic cytokine mainly secreted by macrophages and adipocytes during inflammation but also by muscle in response to contraction, whose receptor is expressed on pancreatic endocrine cells. IL-6 has been shown to enhance insulin secretion either directly through PLC-dependent pathway in β cells (34) or indirectly by the stimulation of L and α cells-secreted GLP-1 (35). However, the regulation of glucose homeostasis by IL-6 could be context dependent and species specific (36). In humans, acute physiological elevations of IL-6 (e.g., during exercise) delays gastric emptying (in GLP-1-independent manner) and reduces insulin secretion (in a GLP-1-dependent manner). These two actions have opposing effects on glucose tolerance, leading to an overall improvement in healthy subjects and no change in mild T2D patients (36, 37).

In 2016, Galgani et al. described that IL-1b-dependent chemokine IL-8 (physiologically secreted by macrophages, endothelial, and epithelial cells) was associated with *in vivo* insulin secretion rates during an oral glucose tolerance test in healthy humans, even if no effect of recombinant human IL-8 on GIST in isolated mice islets was observed (38). IL-8 signaling involves [Ca2+]i (39, 40), but it is not still clear if it may include a direct action on insulin secretion.

Taken together these evidences suggested that inflammation-related molecules could play not only a role in immunity but also a direct role in maintaining glucose homeostasis. A comprehensive knowledge of their physiological roles, beside the pathological ones, would help to understand better many clinical conditions associated to inflammation, as T1D, T2D, and obesity, and to select the best-targeted therapies.

## Cytokine Pathways as Potential Pharmacologic Target to Affect Glucose Homeostasis and Autoimmunity

Immunomodulatory strategies to target diabetes are a growing topic in both preclinical and clinical studies. Regarding the efficacy of these treatments, there is no clear common feeling, and the scientific opinions span from enthusiastic (41) to more skeptical (42). From our perspective, the topic is extremely relevant and we have also contributed something to the immunomodulation of the cytokine pathways for the protection/improvement of the pancreatic islet function within the context of prevention of autoimmune progression (15) and after islet transplantation (43–45). The basic promise is the fact that the glucose homeostasis is fine-tuned by tissue resident immune cells (e.g., macrophages, neutrophils) in most of the metabolic active tissues (e.g., adipose and liver tissues). These cells absolve a crucial role in preserving metabolic homeostasis through cross talk with metabolic tissues in the peripheral system. Alteration of this process may lead to metabolic dysregulation at large as well as many associated diseases. Tissue resident immune cells, as described above, have also been suggested to play a role within the endocrine pancreas. The modulation of their function in T1D in term of cytokines production is important to determine their double role as regulator of immunoreaction and glucose homeostasis. In fact, alongside the benefit obtained from the modulation of the immune system, an additional benefit can be obtained from the inhibition of cytokines depending on their direct role in insulin secretion. If we assume that an exposure to pro-inflammatory cytokines could lead to β cell exhaustion and death due to the direct insulin overstimulation, the honeymoon phase in T1D is a relevant time window to inhibit the acute islet inflammation and delay β cell deregulation and death. Similarly, anti-inflammatory treatment may be useful to preserve the described residual β cell function in long standing T1D by inhibiting the chronic islet inflammation (46). Since pro-inflammatory cytokines are expressed in the pancreatic islet, although the relative expression of innate and adaptive cytokines differs between models (47), the therapeutic efficacy of the immunomodulatory strategies against innate immune cells in T1D is under investigation (**Table 1**). In 2008, etanercept, an anti-TNFα compound, was clinically tested in children newly diagnosed with T1D. A randomized double blind intervention clinical trial (NCT00730392) with etanercept resulted in lower A1C and increased endogenous insulin production, suggesting preservation of beta-cell function in the T1D treated patients. Unfortunately, this study was performed in a small cohort of 18 patients and a larger study is needed to further explore safety and efficacy (48). A second candidate was IL-1. Two randomized double-blind clinical trials with different IL-1 antagonists [canakinumab (NCT00947427) and anakinra (NCT00711503)] were performed on a cohort of 69 patients with new onset of T1D for each trial. Stimulated C-peptide concentrations and percentages of HbA1c did not differ between intervention-treated and placebo-treated patients (49). Interleukin-6 inhibition based treatment was evaluated in T1D clinical trial as additional player of the T1D inflammatory milieu. The EXTEND study is a phase II, multicenter, double blind, placebo-controlled, randomized trial aimed to test the tocilizumab, an IL-6 receptor inhibitor, in a cohort of 30 adults with type 1 diabetes (NCT02293837). As of now no updates are available, and the EXTEND study is expected to report in 2020 (50). As an additional player, we recently investigated on the role of the IL-8-CXCR1/2 pathway in the T1D. Indeed, based on our encouraging preclinical data on the role of CXCR1/2 inhibition in NOD mice (52), we investigated the role of ladarixin, a CXCR1/2 inhibitor, in the preservation of β cell function and slow-down of the progression of T1D in a phase II, multicenter, double-blind study that involved 76 patients with new-onset (NCT02814838). Preliminary results suggest that a subgroup of patients could be beneficial from ladarixin treatment: after 26 weeks from first drug administration, they showed a delay in the c-peptide decline compared to the placebo arm. More detailed analyses are ongoing to clearly define the role of IL-8 pathway inhibition on the beta cell pathophysiology (51).

**Table 1:** State of the art of clinical anti-cytokine and anti-chemokine receptor compounds in T1D patients.

Drug name	Molecule type	Target	Mechanism of action	Effects on beta cell	Trial		Ref
Etanercept	Recombinant fusion protein	TNFα	Soluble TNFα Receptor fusion protein able to bind and block TNFα activity	Indirect effect: lower A1C and increased endogenous insulin production	NCT00730392 (phase II)	Closed 01/2008	(48)
Canakinumab	mAb	IL-1	Neutralization of IL-1 signaling	Direct effect: blocks direct and hyper-glycemia mediated beta cell toxicity	NCT00947427 (phase II)	Open	(49)
Anakinra	Recombinant protein	IL-1	IL-1 competitive receptor antagonist		NCT00711503 (phase II)	Closed 06/2012	(49)
Tocilizumab	mAb	IL-6R	IL-6 Receptor inhibitor	Unknown	NCT02293837 (phase II)	Closed 08/2020	(50)
Ladarixin	Small molecule	CXCR1/2	CXCR1/2 allosteric not competitive inhibitor	Indirect effect: reduction recruiting CXCR1/2+ cells and delay in the c-peptide decline compared to the placebo arm	NCT02814838 (phase II)	Closed 10/2019	(51)

Monoclonal antibody (mAb).

## Conclusion

**Figure 1:**
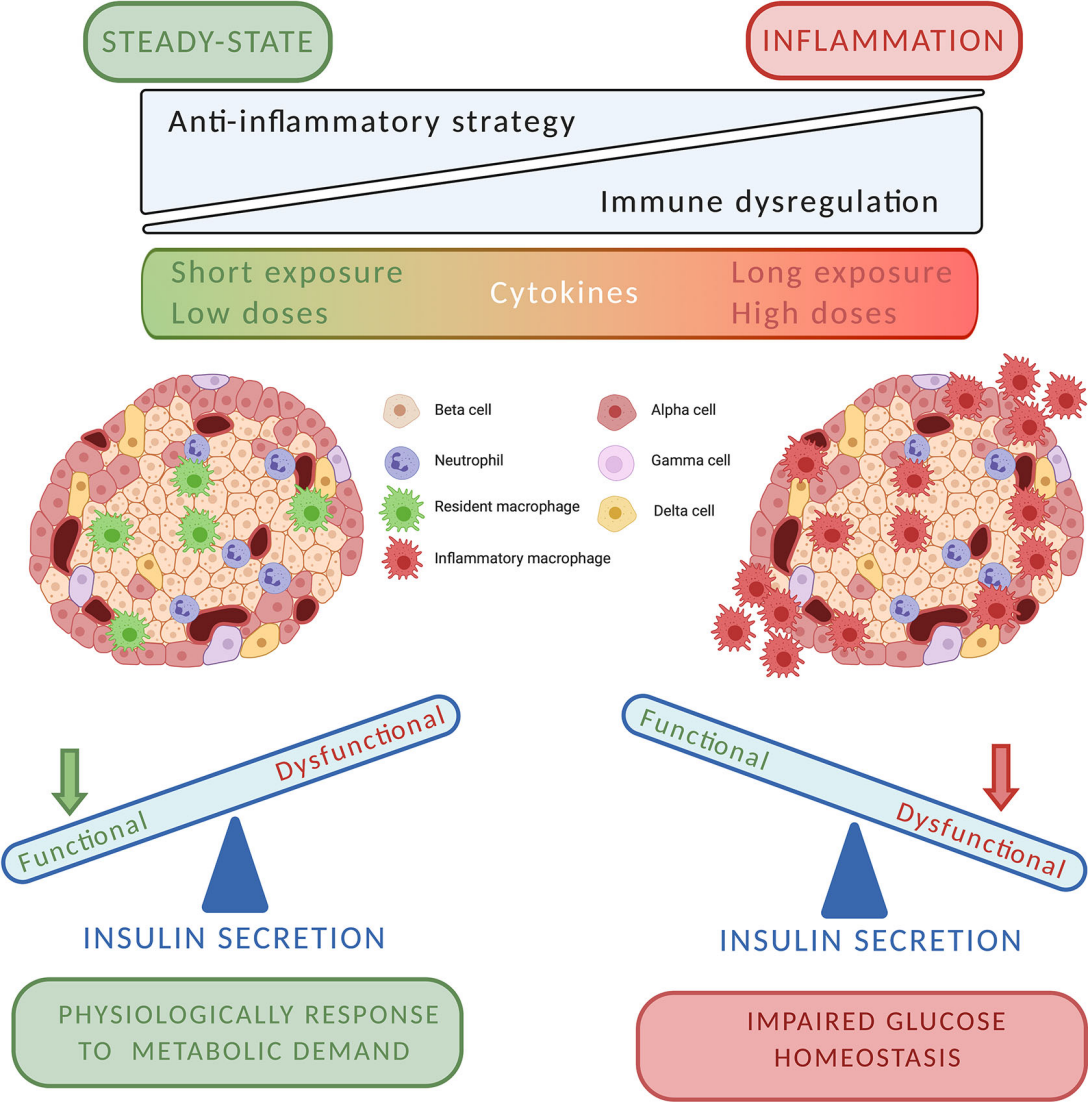
The putative two–faces of cytokines in pancreatic islet milieu.

In this review, we support the idea that T1D disease should not be depicted anymore as an exclusively T cell-mediated autoimmune disease. In fact, there are increasing evidences of a strong involvement of innate immunity in T1D pathogenesis, as innate immune cells appear responsible for both the impaired islet homeostasis and the inflammatory damage. To understand the clinical potential of different anti-inflammatory treatments, we need in the future to study the role of innate immunity in the physiology of islet, paying particular attention to their impact on glucose homeostasis. Findings demonstrate that cytokines can modulate indirectly or directly insulin secretion acting on the same signaling mechanisms of glucose. Moreover, macrophages and neutrophils physiologically reside in the pancreatic endocrine compartment and participate in tissue homeostasis and beta cell function. On the other hand, innate immunity cell and mediator can trigger the inflammatory reaction promoting the immune response. As pictured in **Figure 1**, cytokines could be physiological and pathological factors. The equilibrium between these two actions is relevant for the pathogenesis of T1D, and more generally for islet damage. It is plausible that cytokines could play a role in the physiology of insulin secretion contributing to islet response to metabolic demand. However, under a strong cytokine “pressure” (as determined by long-time and/or high concentration exposure) islet can develop dysfunctional responses, which result in both impaired insulin secretion and insulitis development. In this perspective, cytokines pathways could represent potential therapeutic targets to control efficiently glucose metabolism and to prevent autoimmune damage. Concordantly, the combination of immunomodulatory strategies against both innate and adaptive immunity should be tested in the next future, as they can be more efficient in preventing or delaying T1D.

## Author Contributions

AC, ED, FC, and LP conceived the study, wrote the manuscript, and contributed to the discussion. All authors contributed to the article and approved the submitted version.

## Conflict of Interest

The authors declare that the research was conducted in the absence of any commercial or financial relationships that could be construed as a potential conflict of interest.
